# The Brain in Cross-Cultural Adjustment: A Pilot Study of Japanese Expatriates Living in the United States

**DOI:** 10.3390/brainsci15060617

**Published:** 2025-06-07

**Authors:** Keisuke Kokubun, Kiyotaka Nemoto, Yoshinori Yamakawa

**Affiliations:** 1 Graduate School of Management, Kyoto University, Kyoto 606-8501, Japan; 2 Department of Psychiatry, Institute of Medicine, University of Tsukuba, Tsukuba 305-8577, Japan; 3 Institute of Innovative Research, Institute of Science Tokyo, Meguro, Tokyo 152-8550, Japan; 4 ImPACT Program of Council for Science, Technology and Innovation, Cabinet Office, Government of Japan, Chiyoda, Tokyo 100-8914, Japan; 5 Office for Academic and Industrial Innovation, Kobe University, Kobe 657-8501, Japan; 6 Brain Impact, Kyoto 606-8501, Japan

**Keywords:** cultural adjustment, expatriates, gray matter volume, triple network

## Abstract

**Background/Objectives**: With the globalization of companies, the cross-cultural adjustment of expatriates working overseas is becoming an increasingly important topic. However, little research has been carried out on the brain, which is the source of the ability to adapt. **Methods**: Therefore, we conducted a pilot study on 10 expatriates working for Japanese local subsidiaries in the United States to analyze the relationship between their gray matter volume (GMV) measured by the Gray Matter Brain Healthcare Quotient and their cross-cultural adjustment and lifestyle. **Results**: As a result, in a partial correlation analysis controlled for demographic variables, there was a significant correlation between whole-brain GMV and general adjustment. A relationship was also shown between the local GMV of the default mode network and central executive network and interaction adjustment. **Conclusions**: This is the first pilot study to clarify the relationship between expatriates’ brain structure and cross-cultural adjustment, suggesting the effectiveness of a biological approach in cross-cultural adjustment research.

## 1. Introduction

As companies globalize, expectations of expatriates are increasing. Learning ability is often cited as a factor in expatriates’ failure or success in adapting to a new culture. Spreitzer et al. [1] argue that learning from significant changes and experiences is important for successful expatriation because learning is the primary driving force in cultural adjustment [2]. Learning does not simply mean solving problems [3] or passively accepting information [4]. Learning refers to people reconstructing themselves in response to external changes and acquiring new perceptions of the world through a cycle of concrete experiences, reflective observation, abstract conceptualization, and active experimentation [2,4]. Kolb [2] (p. 31) states that ‘[Learning] involves the integrated functioning of the total organism—thinking, feeling, perceiving, and behaving’, arguing that learning goes beyond descriptions of socialization and change to be a holistic process of adjustment to the environment. In many cases, cultural adjustment refers to three interrelated facets: (1) general adjustment with various aspects of the environment in the host country; (2) interaction adjustment with locals in work and non-work settings; and (3) work adjustment with specific expectations and standards in the work context, based on the degree of psychological comfort and familiarity that expatriates feel with various aspects of the host country culture [5,6].

Yamazaki & Kayes [7] applied Kolb’s learning theory to the study of cross-cultural adjustment, attempting to build a theoretical framework on expatriate adjustment and their learning styles through the perspective of how expatriates learn from experience. The results revealed that as Japanese managers spend more time in the United States, they become more concrete and proactive in their learning styles, and that the learning styles of expatriates change according to cultural demands, and the patterns of change do not necessarily reflect those of US managers. This suggests that Japanese managers do not directly assimilate into the US culture but develop a specialized mode of adjustment to the host culture. Therefore, Yamazaki and Kays [8] argued that what Kolb [2] calls adaptive flexibility is a key factor in successful cross-cultural adjustment. Cross-cultural adjustment is a multifaceted phenomenon [9], and therefore expatriate adjustment can be considered to consist of affective, cognitive, and behavioral dimensions [10]. In this regard, the scope of research on expatriate adjustment needs to be developed in terms of cognitive and behavioral concerns, rather than emphasizing only the affective aspects, which is the main tendency in the literature on expatriate management [7,8].

Neuroscience supports Kolb’s learning theory in the areas of novelty, holistic learning, active learning, and emotional connections [11]. Experiential learning engages both prefrontal and brainstem activity [12], integrating different neural networks [13], resulting in connections between multiple memory pathways and abstract concepts [14]. For example, students’ personal explanations and demonstrations of concepts through multiple modalities result in higher retention [15]. And for expatriates, consistent with Kolb’s learning theory, cultural differences constitute a stimulus for the experiential learning environment, so learning flexibility serves to enhance expatriate effectiveness [16]. This conceptual overlap between experiential learning and intercultural adjustment suggests the validity of applying neuroscience to intercultural adjustment research.

A recent empirical study, using expatriate data from India and China, showed that the negative effect of cultural novelty on general adjustment is mitigated by emotional stability [17]. Relatedly, it is suggested that training methods for expatriates be developed that draw on insights from Kolb’s experiential learning theory [18,19]. However, the aspect of the “total organism” that Kolb [2] takes as the basis of learning has rarely been the subject of research on cross-cultural adjustment. Therefore, to the authors’ knowledge, there have been no studies that have addressed intercultural adjustment using neuroscientific methods. The representative brain networks that neuroscience deals with are three networks that control rational judgment, emotion control, other recognition, self-recognition, and behavior evaluation. Of these, the central executive network (CEN), consisting of the dorsolateral prefrontal cortex and posterior parietal cortex, is crucial for the active retention and manipulation of information, such as working memory, attention, problem solving, decision-making, and self-awareness [20,21,22]. The saliency network (SN) includes the ventrolateral prefrontal cortex (VLPFC), anterior insular cortex, and anterior cingulate cortex (ACC) [23], and responds to subjective salience [24]. The SN also functions as a switch between the CEN and default mode network (DMN) for attention and flexible cognitive control [25,26]. Meanwhile, the DMN includes the posterior cingulate cortex (PCC) and parts of the frontal lobe, the medial prefrontal cortex (MPFC), and the posterior temporal lobe region around the temporoparietal junction (TPJ), including the inferior parietal lobe (IPL) [27,28]. The DMN is involved in engaging various domains of cognitive and social processing [27] and in understanding the mental states of others [29]. The interactions between these networks are involved in self-regulation and cooperation with others in daily life [30,31,32,33].

The triple network may be relevant for cross-cultural adjustment. The DMN may be involved in modeling the emotions, intentions, and characteristics of others to simulate, explain, and predict their behavior [34,35]. The SN may also function as a general motivational system to encode the reward/punishment properties of social choices and outcomes with reference to social principles [36,37,38]. Finally, the CEN may be involved in integrating information encoded in the DMN and SN to suppress self-interest and immediate gratification and optimize social behavior [39,40]. Thus, a recent meta-analysis identified these networks as brain regions that mediate social cognition, motivation, and cognitive control and are consistently involved in diverse social interactions [41]. In studies focusing on brain structure, the degree of mentalizing impairment [42,43] and apathy in Alzheimer’s dementia patients [44] were correlated with the level of atrophy in the DMN region. On the other hand, the gray matter degeneration of the SN in frontotemporal dementia was associated with a loss of emotional empathy [45]. Furthermore, a meta-analysis showed a moderate positive relationship between the volume or thickness of the prefrontal cortex and performance on executive function tasks in healthy adults, consistent with the role of the CEN revealed by functional neuroimaging studies [46].

Lifestyle is also related to cross-cultural adjustment and the brain. It is also important to note that adapting to a different culture does not necessarily mean living a healthy lifestyle, another aspect that previous cultural adjustment research has overlooked. Food and sleep are related to expatriates’ health and performance [47,48]. For example, a survey conducted by Truman et al. showed that 36% of expatriates living abroad had sleep disorders, which worsened their performance and interpersonal relationships [47]. The issue of food is not easy either. A Fujimoto study showed that diabetes is much more common among Japanese Americans than the general Japanese and American populations [48]. This means that adjusting to the local lifestyle can be harmful to health. At the same time, lifestyle, including health and recreation, is related to the success or failure of expatriates’ work and tasks [49,50]. For example, a study of 118 culturally diverse expatriates working in Europe showed that conflicts between personal and work life were related to their health concerns [51]. Furthermore, the fact that these problems affect not only expatriates’ intercultural adjustment but also their brain structure can be inferred from studies of domestic residents showing that lifestyle disorders are associated with an atrophy of gray matter volume (GMV) [52,53,54].

However, to our knowledge, there have been no studies that have addressed the relationship between the brain structure centered on these triple networks and cross-cultural adjustment or lifestyle. Therefore, in this study, we contribute to the development of related research by clarifying the characteristics of the brain structure centered on the triple network in expatriates who are cross-culturally adjusting. The sample was Japanese expatriates residing in the United States. Previous studies have shown differences in mindset between the United States and Japanese cultures [55,56,57]. Such differences between home and host cultures may pose particular challenges for expatriates in adapting [58]. Indeed, there is evidence that Japanese expatriates have great difficulty interacting with their US colleagues [59,60]. These findings suggest that Japanese and US cultures provide fertile ground for understanding the differences encountered in cross-cultural adjustment and therefore that biological mechanisms need to be elucidated.

In this study, brain structure was measured using GMV calculated from brain images using the Gray Matter Brain Healthcare Quotient (GM-BHQ) developed by Nemoto et al. [61]. The GM-BHQ has been approved as an international standard by the standardization organization International Telecommunication Union Telecommunication Standardization Sector (ITU-T) as a “numerical index representing the physical characteristics of the brain that indicate health-related conditions” (approval number: ITU-TH.861.0). Therefore, using the GM-BHQ has an advantage when considering the internationalization of future research. Recently, Otsuka et al. reported a pilot study using the GM-BHQ to show that a high level of understanding of different genders and origins is related to a larger GMV of triple networks [62]. In addition, self-reported data from a questionnaire survey was used for lifestyle and cultural adjustment in the current research. Although self-reporting has the disadvantage of being susceptible to social desirability and recall bias, it has the advantage of being low-cost and relatively easy to use, and allows us to investigate behaviors that may not be observable by other methods [63]. Because the lifestyle and intercultural adjustment of expatriates surveyed in this study are diverse, we decided that a format in which participants themselves recall and self-report would be the most appropriate.

## 2. Materials and Methods

### 2.1. Participants

The sample size required to perform correlation analysis at a significance level of 10% and power of 80%, with an effect size of r = 0.7, which corresponds to a “strong correlation” according to Schober et al. [64], was calculated as 9 samples using G*Power 3.1.9.7. A total of 10 people (9 men and 1 woman) participated in the study after public recruitment through web advertisements, with a mean age of 44.9 ± 7.3 years. Participants completed online questionnaires from October to November 2023, and MRI images were obtained at a clinic in Silicon Valley, USA, from February to March 2024. According to self-report, none of the participants had a history of neurological, psychiatric, or other medical conditions that could affect the central nervous system. All methods were carried out in accordance with relevant guidelines and regulations, all participants provided written informed consent before participation, and anonymity was maintained. This study was approved by the Institute of Science Tokyo’s Brain Information Cloud (Ethics Committee for Human Research: Permit Number 2023137) Ethics Committee. Table 1 and Table 2 show information on the various variables of the participants.

### 2.2. MRI Data Acquisition

A 3 Tesla MRI scanner (MAGNETOM Prisma, Siemens, Munich, Germany) with a 32-channel head array coil, three-dimensional (3D) T1-weighted magnetization-prepared rapid acquisition gradient echo pulse sequence, and spin-echo echo-planar imaging (SE-EPI) with generalized autocalibrated partially parallel acquisition (GRAPPA) was used for magnetic resonance imaging (MRI) data collection and structural imaging. The following parameters were used: repetition time (TR): 1900 ms; echo time (TE): 2.52 ms; inversion time (TI): 900 ms; flip angle: 9°; matrix size: 256 × 256; field of view (FOV): 256 mm; and slice thickness: 1 mm.

### 2.3. MRI Data Analysis

T1-weighted images were segmented into gray matter (GM), white matter (WM), and cerebrospinal fluid (CSF) using Statistical Parametric Mapping 12 (SPM12; Wellcome Trust Center for Neuroimaging, London, UK) in MATLAB R2020b (MathWorks Inc., Sherborne, MA, USA) and SPM12 prior probability templates. The segmented GM images were then spatially normalized using diffeomorphic anatomical registration using the exponential lie algebra (DARTEL) algorithm [65], which included incorporating a modulation step into the preprocessing model and smoothing images using a Gaussian kernel with a full width at half maximum (FWHM) of 8 mm. The smoothed GM images were then converted to proportional GM images by dividing by the intracranial volume (ICV) and used to create mean and standard deviation (SD) images. By averaging this information and the local GM quotients using the automatic anatomical labeling (AAL) atlas [66], the GM-BHQ was created with a mean of 100 and an SD of 15 points.

In previous studies, the whole-brain GM-BHQ has been related to dietary balance [52], lifestyle [53], and diversity understanding [62]. These studies were conducted in healthy middle-aged adults, not elderly people or people with underlying diseases, so it is expected that using the same index will add depth to the interpretation of the study. Our study, which focuses on GMV, a function of surface area and cortical thickness, is more comprehensive than studies using other neural biomarkers such as functional MRI (fMRI), which is excellent for examining brain responses [67], and cortical thickness, which is suitable for observing genetic influences [68]. Table 3 shows how the CEN, SN, and DMN were aggregated from the AAL atlas labels.

### 2.4. Psychological Test

#### 2.4.1. Cultural Adjustment

Adopted from Black and Stephens [69], a total of 14 items make up the following three facets: general adjustment (7 items, e.g., “Living conditions in general”, “Housing conditions”); interaction adjustment (4 items, e.g., “Socializing with host nationals”); work adjustment (3 items, e.g., “Specific job responsibilities”). All items are rated on a 7-point Likert scale (1 = not adjusted at all; 7 = very well adjusted), and the value of each variable is calculated by the average of the items.

#### 2.4.2. Lifestyle

As a variable representing lifestyle, we used the total score of BHQ Actions scale, which consists of seven subsets: “Healthcare” (one item, “To what extent are you interested in your own health?”), “Social life” (one item, “How many social relationships do you have?”), “Learning” (one item, “How often have you engaged in hobbies or learning in the past year?”), “Exercise” (one item, “How many times a week do you exercise for 30 min or more?”), “Environment” (one item, “How much time do you have per week to go outside and experience nature?”), “Rest” (five items, e.g., “I fall asleep easily”), and “Diet” (fifteen items, e.g., “Ate fish 1 or more times per week”). For details of each scale and the questionnaire, please refer to Kokubun, Nemoto et al. [70].

#### 2.4.3. Control Variables

Age (years old), educational background (years), period of stay in the US (months), body mass index (BMI), and sex (male = 1; female = 0) were used as control variables. Because these variables are correlated with each other, their individual inclusion can lead to multicollinearity problems in the regression model [71]. Therefore, we applied principal component analysis (PCA) to the control variables, a method often adopted to avoid this problem. As a result of the principal component analysis, only the first principal component (contribution rate: 47.462%) had an eigenvalue exceeding 1. Furthermore, the scree plot showed that the eigenvalues varied from 2.373 to 0.996, 0.862, 0.586, and 0.183, indicating a large drop between factor 1 and factor 2. These results indicate that it is appropriate to consider the control variables as a one-factor structure. Therefore, in order to maintain the degrees of freedom of the regression model, we decided to use the first principal component scores of the above demographic variables as indicated in Table 4 as control variables, rather than using them individually.

### 2.5. Data Analysis

Correlation and partial correlation analyses were performed between the whole-brain and the regional GMVs of the CEN, SN, and DMN, the three variables representing cross-cultural adjustment, and one variable representing lifestyle. Given that this is a small-sample pilot study, statistical significance was defined as a 0.1 < *p*-value. This is because the purpose of a pilot study is to obtain information about data that may be collected in a future larger study, so it is more rational to avoid making a Type II error than to make a Type I error. This decision has been supported by previous research [72] and therefore many recent pilot studies have also adopted a *p*-value of 0.1< for statistical significance, e.g., [73,74,75]. Bonferroni tests for multiple comparisons were performed for each whole-brain and regional GMV, and the criterion for significance was set at 0.025 < *p*-value (=0.1/4). That is, because 4 tests (3 for intercultural adjustment and 1 for lifestyle) were performed with a significance level of *p* < 0.1, *p* < 0.025 was used as the criterion for significance in multiple comparisons. Correlation coefficients were also evaluated in terms of effect size, with r = 0.7 corresponding to the “strong correlation” of Schober et al. [64] as the criterion. In addition, correlation coefficients were also evaluated with confidence intervals corresponding to *p* < 0.1. All statistical analyses were performed using IBM SPSS Statistics Version 28 (IBM Corp., Armonk, NY, USA).

## 3. Results

**Figure 1:**
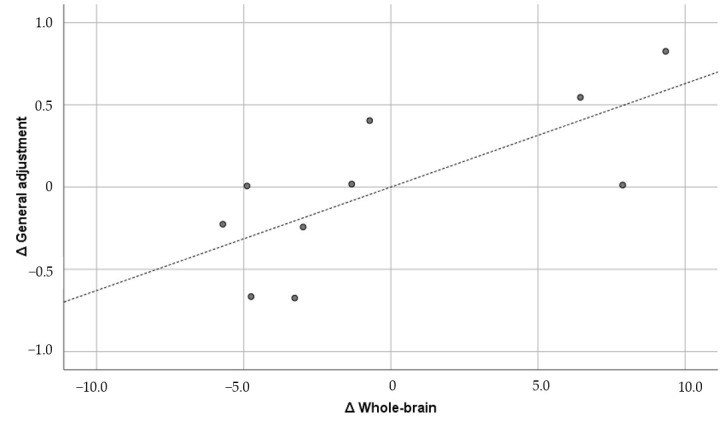
Whole brain and general adjustment (r = 0.733). ∆: Residuals from a regression with the control variables as independent variables. The dashed line is the regression line, and the dots are the scores of each participant.

**Figure 2:**
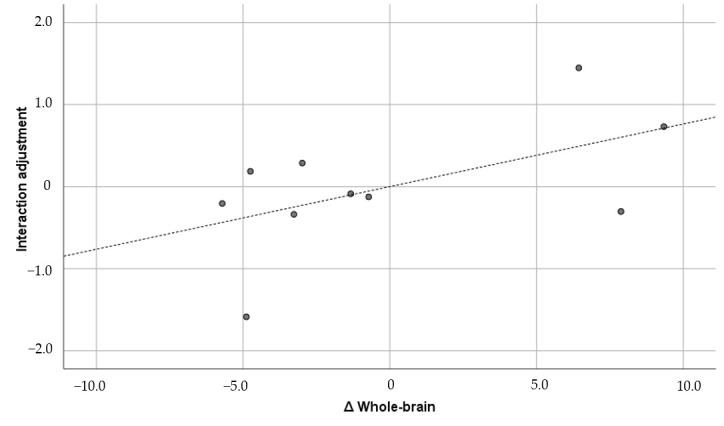
Whole brain and interaction adjustment (r = 0.554). ∆: Residuals from a regression with the control variables as independent variables. The dashed line is the regression line, and the dots are the scores of each participant.

**Figure 3:**
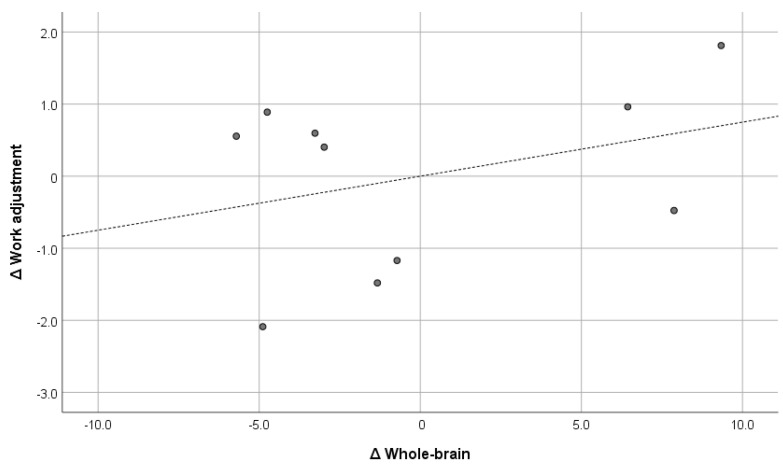
Whole brain and work adjustment (r = 0.342). ∆: Residuals from a regression with the control variables as independent variables. The dashed line is the regression line, and the dots are the scores of each participant.

**Figure 4:**
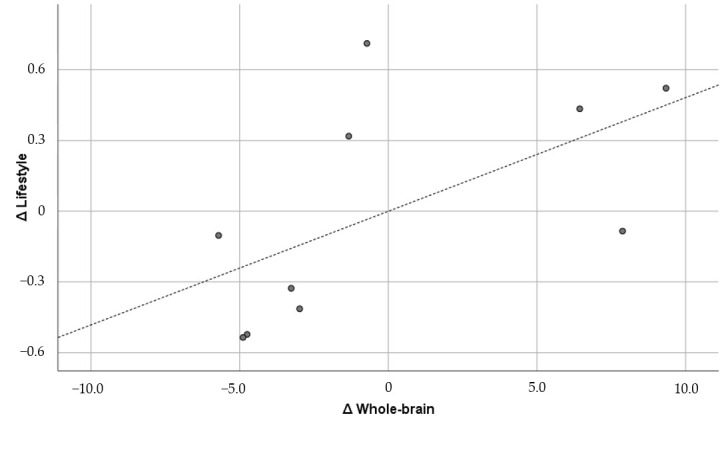
Whole brain and lifestyle (r = 0.593). ∆: Residuals from a regression with the control variables as independent variables. The dashed line is the regression line, and the dots are the scores of each participant.

The figures below the diagonal show the Pearson correlation coefficients, and the figures above the diagonal show the Pearson partial correlation coefficients in Table 5. The former is the uncontrolled correlation coefficient, and the latter is the correlation coefficient controlled by the first principal component. The numbers in parentheses under the correlation coefficients indicate the 90% confidence interval. The correlation coefficients did not show any significant correlation between GMV and the psychological variables. Therefore, the results of the partial correlation coefficients are shown below. GMV was positively and significantly correlated with general adjustment (r = 0.733; *p* = 0.0248) and lifestyle (r = 0.593; *p* = 0.092), but it was not significantly correlated with interaction adjustment (r = 0.554; *p* = 0.122) or work adjustment (r = 0.342; *p* = 0.367). Of these, the correlation with general adjustment met the Bonferroni multiple comparison significance criterion of *p* < 0.025. Furthermore, only general adjustment exceeded r = 0.7, which corresponds to the “strong correlation” of Schober et al. [64]. The confidence interval was also well above zero (0.304, 0.914). For comparison, Figure 1, Figure 2, Figure 3 and Figure 4 show the results of all these correlations in scatter plots. (In multiple comparisons, only the general adjustment in Figure 1 was significant, but other correlations are also shown to make the differences easier to see).

**Figure 5:**
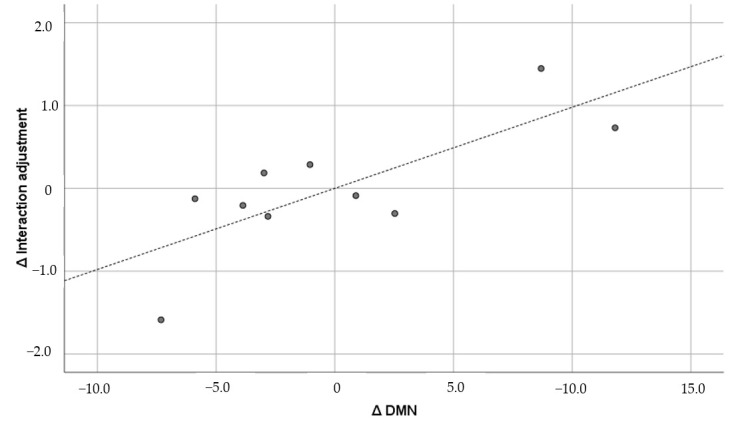
DMN and interaction adjustment (r = 0.771). ∆: Residuals from a regression with the control variables as independent variables. The dashed line is the regression line, and the dots are the scores of each participant.

**Figure 6:**
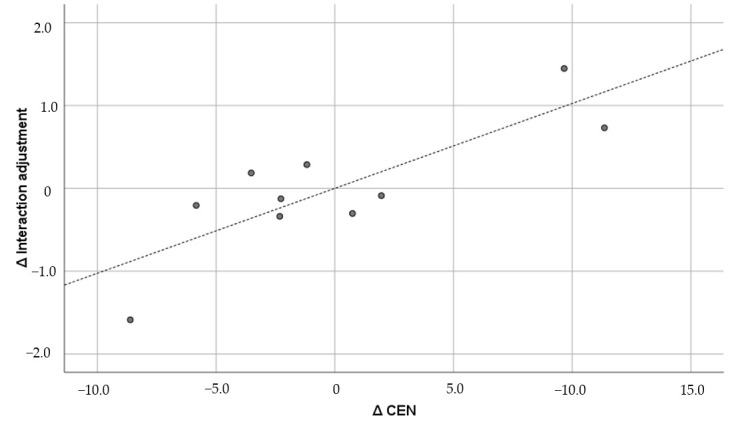
CEN and interaction adjustment (r = 0.826). ∆: Residuals from a regression with the control variables as independent variables. The dashed line is the regression line, and the dots are the scores of each participant.

Looking at the results of local GMVs, DMN was positively and significantly correlated with general adjustment (r = 0.630; *p* = 0.069), interaction adjustment (r = 0.771; *p* = 0.015), and work adjustment (r = 0.614; *p* = 0.079). CEN was positively and significantly correlated with general adjustment (r = 0.691; *p* = 0.039), interaction adjustment (r = 0.826; *p* = 0.006), and lifestyle (r = 0.660; *p* = 0.053). SN was positively and significantly correlated with general adjustment (r = 0.682; *p* = 0.043), interaction adjustment (r = 0.663; *p* = 0.052), and lifestyle (r = 0.593; *p* = 0.093). However, after multiple comparisons using the Bonferroni test, only two correlations met the *p* < 0.025 criterion: between DMN and interaction adjustment, and between CEN and interaction adjustment. These two pairs were the only ones that exceeded r = 0.7, the “strong correlation” [64]. The confidence intervals for DMN (0.381, 0.928) and CEN (0.503, 0.947) were also significantly greater than zero. Figure 5 and Figure 6 show the correlations that survived multiple comparisons among the local GMVs. (Here, we only show correlations between DMN and CEN and interaction adjustment that were significant using the Bonferroni-corrected criterion.)

## 4. Discussion

The results of this study suggest that the whole-brain GMV of Japanese expatriates living in the United States is related to general adjustment in a cross-cultural environment. This is reasonable in light of previous research showing that whole-brain GMV is positively related to cognitive ability [76] and social performance [77] and mediates the association between psychological distress and job satisfaction [78]. In other words, high cognitive and social abilities, backed by large GMV, may facilitate life in the host country and promote general adjustment, which in turn may suppress the consumption of brain resources and maintain high GMV. Such reasoning is consistent with discussions of neuroplasticity and the view of the brain as a resource that can be changed depending on the environment [79,80]. Although intercultural adjustment tends to focus on the psychological aspects, the results of this study suggest that, as some researchers have previously argued [5,6], it is necessary to consider adjustment from a comprehensive perspective, focusing on biological factors.

Analysis by network suggests that the DMN and CEN play an important role in interaction adjustment. On the other hand, no significant correlations were found for the SN, which is active when switches between the DMN and CEN are necessary. Considering that the DMN is related to reading other people’s emotions and predicting their behavior [34,35], and the CEN is related to suppressing self-interest and optimizing behavior [39,40], the results of this study that these networks are related to intercultural adjustment, especially interaction adjustment, are reasonable. In addition, this result is consistent with Otsuka et al. [62], who showed that both the DMN and CEN are necessary for understanding and adopting diversity. Otsuka et al. argued that in cooperation with diverse entities, the ability to empathize with others, that is, the DMN alone, does not benefit the individual and therefore the relationship will not last, and thus the CEN is necessary to understand the strengths of others and bring out their potential abilities [62]. The results of this study can also be interpreted as supporting this claim.

On the other hand, no significant correlations were found for the SN which is involved in switching between the DMN and CEN [25,26]. It met the 0.05 criterion but not the more stringent 0.025 criterion. As a pilot study, this correlation is indicative of a possible relation, but more research will need to be conducted to determine whether this is a reliable relation. Similarly, work adjustment was not correlated with any of the three networks. This can be interpreted if improvements in general adjustment and interaction adjustment precede improvements in work adjustment. Nakahara argues that the “concrete experiences” included in Kolb’s experiential learning model refer to interactions that occur when learners interact with other people, artifacts, and the environment, and therefore are value-neutral and do not include business or management elements [81]. Consistent with this, Takeuchi et al.’s study of Japanese expatriates found a significant positive correlation between spillover effects from non-work variables to work variables, but not from work to non-work variables [82]. Similarly, Bell and Harrison argued that interactional adjustment is the most fundamental aspect of the three, as both work and general adjustment are influenced by interactions with local people [83]. A meta-analysis by Bhaskar-Shrinivas et al. also showed that low levels of general adjustment affect work performance [84]. Another possibility for the lack of correlations with work adjustment is that the measure is about specific job responsibilities and not about adjustment per se. Perhaps this measure does not capture anything about cross-cultural adjustment and therefore the networks would not be as involved as with the other measures of adjustment. Thus, consistent with previous studies, the results of this study suggest that the expatriate environment and interactions with others may change the relationship between the brain and general and interaction adjustment, which may in turn change work adjustment.

For lifestyle variables that did not achieve the significance level in multiple comparisons, the contrast with general and interaction adjustment, which showed a significant correlation with GMV, is suggestive. This is because general and interaction adjustment overlap with lifestyle variables and include adjustments to diet and relationships. Previous studies have suggested not only that expatriate lifestyles tend to be unhealthy [47] but also that adjustment may lead to unhealthiness [48]. Thus, the results of this study suggest that in a cross-cultural environment, achieving a healthy lifestyle is more difficult than adjusting to diet or relationships, and is achieved through more experiential learning.

The results of this study suggest that an interdisciplinary approach combining research into the intercultural adjustment of expatriates with brain science may be effective. In today’s world, where globalization is progressing and the performance of expatriates has an increasing influence on the trends of companies, it is hoped that focusing on the brains of expatriates will lead to more effective methods for training and selecting highly adaptable personnel, thereby contributing to the development of companies and improving the happiness of expatriates and local employees alike.

## 5. Limitations

The results of this study have five limitations. First, this is a pilot study with a very small sample size, and therefore its results are highly susceptible to both Type I and Type II errors, and are experimental attempts for which reproducibility is not guaranteed. Second, the sample was mainly Japanese men (9 out of 10) and therefore may bias the findings if there are differences in the brain and cross-cultural adjustment related to origin and gender. Third, data collection spanned two periods and may have been influenced by individual differences in life events during that time. Fourth, the results of this study were obtained through cross-sectional analysis and do not indicate causal relationships. Fifth, this study used self-report measures, so social desirability and recall bias may have influenced the results. In the future, the results of this study need to be verified and developed through longitudinal, interventional, and fMRI brain response studies with a larger sample size and a more objective measure, including participants of various origins and genders.

## 6. Conclusions

In a globalized society, the cross-cultural adjustment of expatriates is a major challenge for many companies. In this study, we conducted a pilot study on 10 Japanese expatriates working for Japanese companies in the United States and found a significant positive correlation between whole brain GMV and general adjustment, and between DMN and CEN GMV and interaction adjustment. These results may be useful in developing methods to more successfully support companies in expanding overseas by applying brain science to the training and selection of expatriates.

## Figures and Tables

**Table 1:** Participant information (frequency).

	N	%
*Sex*		
Male	9	90
Female	1	10
*Occupation*		
Managerial	5	50
Professional and technical	4	40
Sales	1	10
*Accompanying family*		
Accompanied	8	80
Unaccompanied	1	10
Single	1	10
*Pre-departure cross-cultural training*		
Yes	6	60
No	4	40

**Table 2:** Participant information (minimum, maximum, and average value).

	N	Min	Max	Mean	SD
*GMV*					
Whole-brain	10	84.9	105.8	93.9	7.4
DMN	10	86.7	108.6	95.7	6.5
CEN	10	82.0	107.4	94.0	7.4
SN	10	86.6	123.8	101.5	10.7
*Cross-cultural adjustment*					
General adjustment	10	4.7	6.1	5.3	0.5
Interaction adjustment	10	2.8	6.0	4.2	0.8
Work adjustment	10	3.0	7.0	5.2	1.2
Lifestyle	10	2.9	4.1	3.4	0.5
Age (years)	10	32.0	56.0	44.9	7.3
BMI (kg/m^2^)	10	17.9	27.8	22.5	3.1
Length of education (months)	10	16.0	21.0	17.3	1.6
Length of service (months)	10	1.0	6.0	3.7	1.8
Period of stay in the US (months)	10	5.0	54.0	26.6	18.7
Period overseas for work (months)	10	5.0	130.0	43.6	37.5
Period overseas for study (months)	10	0.0	24.0	5.2	7.7
Period overseas for others (months)	10	0.0	74.0	17.2	28.7

SD: standard deviation.

**Table 3:** Network and region correspondence.

Network	AAL Code	Region
DMN	AAL023	Superior medial frontal gyrus (Left)
	AAL024	Superior medial frontal gyrus (Right)
	AAL035	Posterior cingulate gyrus (Left)
	AAL036	Posterior cingulate gyrus (Right)
	AAL061	Inferior parietal lobule (Left)
	AAL062	Inferior parietal lobule (Right)
	AAL067	Precuneus (Left)
	AAL068	Precuneus (Right)
CEN	AAL003	Superior frontal gyrus (Left)
	AAL004	Superior frontal gyrus (Right)
	AAL059	Superior parietal lobule (Left)
	AAL060	Superior parietal lobule (Right)
SN	AAL029	Insula (Left)
	AAL030	Insula (Right)
	AAL031	Anterior cingulate gyrus (Left)
	AAL032	Anterior cingulate gyrus (Right)

**Table 4:** The first principal component scores of the demographic variables.

	Factor 1
Sex (male 1; female 0)	0.770
Age (years)	0.908
BMI (kg/m^2^)	0.714
Length of education (months)	0.595
Period of stay in the US (months)	0.304

**Table 5:** Correlation coefficient.

		1	2	3	4	5	6	7	8
1	Whole-brain		0.865 **	0.854 **	0.892 **	0.733 *	0.554	0.342	0.593 ^†^
			(0.599, 0.959)	(0.571, 0.956)	(0.670, 0.968)	(0.304, 0.914)	(0.002, 0.847)	(−0.259, 0.752)	(0.061, 0.863)
2	DMN	0.830 **		0.965 ***	0.950 ***	0.630 ^†^	0.771 *	0.614 ^†^	0.508
		(0.513, 0.948)		(0.884, 0.990)	(0.837, 0.985)	(0.119, 0.877)	(0.381, 0.928)	(0.093, 0.871)	(−0.062, 0.828)
3	CEN	0.894 ***	0.944 ***		0.938 ***	0.691 *	0.826 **	0.551	0.660 ^†^
		(0.675, 0.968)	(0.818, 0.984)		(0.800, 0.982)	(0.224, 0.900)	(0.503, 0.947)	(−0.002, 0.846)	(0.169, 0.888)
4	SN	0.937 ***	0.884 **	0.946 ***		0.682 *	0.663 ^†^	0.572	0.593 ^†^
		(0.797, 0.981)	(0.648, 0.965)	(0.824, 0.984)		(0.208, 0.897)	(0.175, 0.890)	(0.029, 0.854)	(0.061, 0.863)
5	General adjustment	0.443	0.536	0.490	0.392		0.408	0.052	0.810 **
		(−0.145, 0.800)	(−0.023, 0.840)	(−0.085, 0.820)	(−0.204, 0.776)		(−0.186, 0.784)	(−0.515, 0.587)	(0.466, 0.941)
6	Interaction adjustment	0.227	0.615	0.527	0.293	0.434		0.730*	0.516
		(−0.372, 0.692)	(0.095, 0.871)	(−0.036, 0.836)	(−0.309, 0.728)	(−0.156, 0.796)		(0.298, 0.914)	(−0.051, 0.831)
7	Work adjustment	0.298	0.600 ^†^	0.498	0.467	0.041	0.684 *		0.065
		(−0.304, 0.730)	(0.071, 0.865)	(−0.075, 0.824)	(−0.115, 0.813)	(−0.523, 0.580)	(0.212, 0.897)		(−0.505, 0.596)
8	Lifestyle	0.395	0.452	0.513	0.385	0.810 **	0.518	0.059	
		(−0.201, 0.778)	(−0.134, 0.804)	(−0.055, 0.830)	(−0.212, 0.773)	(0.466, 0.941)	(−0.048, 0.832)	(−0.510, 0.592)	

N = 10; ^†^ *p* < 0.10; * *p* < 0.05; ** *p* < 0.01; *** *p* < 0.001. BMI: body mass index (BMI, kg/m^2^). The figures below the diagonal are Pearson’s correlation coefficients. The figures above the diagonal are Pearson’s correlation coefficients controlling for demographic variables. In brackets are 90% confidence intervals.

## Data Availability

The data presented in this study are available on request from the corresponding author due to the need to protect the privacy of participants.
